# Nutrition Recommendations and the Children’s Food and Beverage Advertising Initiative’s 2014 Approved Food and Beverage Product List

**DOI:** 10.5888/pcd12.140472

**Published:** 2015-04-23

**Authors:** Rebecca M. Schermbeck, Lisa M. Powell

**Affiliations:** Author Affiliation: Lisa M. Powell, Division of Health Policy and Administration, School of Public Health and Institute for Health Research and Policy, University of Illinois at Chicago, Chicago, Illinois.

## Abstract

We compare the Children’s Food and Beverage Advertising Initiative’s (CFBAI’s) April 2014 list of food and beverage products approved to be advertised on children’s television programs with the federal Interagency Working Group’s nutrition recommendations for such advertised products. Products were assessed by using the nutrients to limit (saturated fat, *trans* fat, sugar, and sodium) component of the Interagency Working Group’s recommendations. Fifty-three percent of the listed products did not meet the nutrition recommendations and, therefore, were ineligible to be advertised. We recommend continued monitoring of food and beverage products marketed to children.

## Objective

Children see 10 to 13 food-related advertisements per day on television, half of which air during programs specifically for children (1). The industry-based Children’s Food and Beverage Advertising Initiative (CFBAI) (Appendix) (2) and the government-based Interagency Working Group (IWG) (representing the Federal Trade Commission, the Centers for Disease Control and Prevention, the Food and Drug Administration, and the US Department of Agriculture) (3), have each created voluntary nutrition guidelines for food and beverage products advertised on children’s programs. The CFBAI is a voluntary industry initiative to self-regulate child-directed food and beverage product advertising (4). Although many food and beverage companies participate in the CFBAI, improvement in the nutritional content of advertised food and beverage products has been limited (1,5–7). Effective in 2014, CFBAI member companies adopted uniform nutrition criteria for food and beverage products focusing on nutrition components to encourage and on limits to calories, saturated fat, sugar, and sodium (2). This study compares the nutrition components of the CFBAI’s April 2014 list of food and beverage products (8) approved for advertisement on children’s programming on the basis of CFBAI’s nutrition standards with IWG’s recommendations for nutrients to limit in food and beverage products advertised to children.

## Methods

We assessed nutrition information for the April 2014 CFBAI-approved product list. Nutrition information was collected from company websites, grocery stores, and product information documents that were on the CFBAI website in April 2014. Products were assessed on the basis of IWG Principal B of recommended nutrients to limit (ie, saturated fat, *trans* fat, sugar, and sodium) in foods marketed to children (3).

Products with more than 1 g of saturated fat per reference amount customarily consumed (RACC) or more than 15% of total calories from saturated fats were classified as high in saturated fat. Main dishes or meals with more than 1 g per 100 g of the product or more than 10% of total calories from saturated fats were deemed high in saturated fat; milk, whole eggs, and nuts were exempt. Products that exceeded 0.5 g of *trans* fat per RACC for individual items or for labeled serving size for main dishes or meals were considered high in *trans* fat. Products were high in sugar if they exceeded 13 g of the RACC of total sugar (nutrition facts panels do not distinguish between added versus natural sugars) for individual items and labeled serving size for main dishes and meals. Milk and yogurt were allowed an additional 12.5 g per RACC and 16 g per RACC, respectively, of sugar, and fruits and 100% fruit or vegetable juices were exempted to account for naturally occurring sugar. Products containing more than 210 mg per RACC of sodium for individual items or 450 mg per labeled serving size for main dishes and meals were considered high in sodium. Per the IWG recommendations, an RACC of 30 g or more was adjusted to a 50 g serving size for analysis (3). Products were assessed for each recommended nutrient to limit and were considered ineligible for advertising on children’s programming if the product exceeded any 1 of the limits.

We did not evaluate IWG Principle A recommendations (nutrients to encourage) because of lack of available nutrient information. We created an 11th exempt category for products CFBAI excluded from the nutrient profiling scheme (ie, sugar-free gum).

## Results

In April 2014, CFBAI issued its list of 407 products approved to be advertised on children’s television programs. Several CFBAI-approved products did not meet IWG recommendations in 10 CFBAI product categories plus a category of exempt products (Table). Dairy products appeared on the approved food and beverage list most frequently (n = 141, 35%) followed by grain, fruit, and vegetable products, and items not in other categories that contained 150 calories or less (n = 113, 28%). Products in the latter category included snack crackers, cookies, and gummy candies.

**Table:** Food and Beverage Products Approved by the Children’s Food and Beverage Advertising Initiative (CFBAI) in April 2014 That Do Not Meet Interagency Working Group (IWG) Recommendations for Nutrients to Limit

Products	Foods on CFBAI Product List, N (%)	Number of Products Exceeding IWG Nutrients to Limit				
		Saturated Fat, N (%)	*Trans* fat, N (%)	Sugar, N (%)	Sodium, N (%)	One or More Nutrients to Limit, N (%)
**Juices**	7 (1.7)	0 (0)	0 (0)	3 (42.9)	0 (0)	3 (42.9)
**Dairy products^a^^,^ b ^,^ c**	141 (34.6)	30 (21.8)	0 (0)	49 (34.8)	0 (0)	69 (49.0)
Milks and milk substitutes	0 (0)	0 (0)	0 (0)	0 (0)	0 (0)	0 (0)
Yogurts and yogurt-type products^c^	134 (32.9)	25 (18.7)	0 (0)	49 (36.4)	0 (0)	64 (47.8)
Dairy-based desserts	2 (0.5)	0 (0)	0 (0)	0 (0)	0 (0)	0 (0)
Cheese and cheese products	5 (1.2)	5 (100.0)	0 (0)	0 (0)	0 (0)	5 (100.0)
**Grain, fruit and vegetable products, and items not in other categories^d^ (≤150 calories)**	113 (27.8)	36 (31.9)	0 (0)	71 (62.8)	9 (8.0)	89 (78.8)
Grain products	78 (19.2)	28 (35.9)	0 (0)	38 (48.7)	9 (11.5)	55 (70.5)
Fruit products^d^	0 (0)	0 (0)	0 (0)	0 (0)	0 (0)	0 (0)
Vegetable products^d^	0 (0)	0 (0)	0 (0)	0 (0)	0 (0)	0 (0)
Items not in other categories	35 (8.6)	8 (22.9)	0 (0)	33 (94.3)	0 (0)	34 (97.1)
**Grain, fruit and vegetable products, and items not in other categories^d^ (>150 calories)**	7 (1.7)	4 (57.1)	0 (0)	2 (28.6)	4 (57.1)	4 (57.1)
Grain products	7 (1.7)	4 (57.1)	0 (0)	2 (28.6)	4 (57.1)	4 (57.1)
Fruit products^d^	0 (0)	0 (0)	0 (0)	0 (0)	0 (0)	0 (0)
Vegetable products^d^	0 (0)	0 (0)	0 (0)	0 (0)	0 (0)	0 (0)
Items not in other categories	0 (0)	0 (0)	0 (0)	0 (0)	0 (0)	0 (0)
**Soups and meat sauces**	0 (0)	0 (0)	0 (0)	0 (0)	0 (0)	0 (0)
**Seeds, nuts, and nut butters and spreads^a^**	8 (2.0)	0 (0)	0 (0)	0 (0)	0 (0)	0 (0)
**Meat, fish, and poultry products**	0 (0)	0 (0)	0 (0)	0 (0)	0 (0)	0 (0)
**Mixed dishes**	24 (5.9)	9 (37.5)	0 (0)	0 (0)	24 (100.0)	24 (100.0)
**Main dishes and entrées**	0 (0)	0 (0)	0 (0)	0 (0)	0 (0)	0 (0)
**Small meals**	10 (2.5)	6 (60.0)	0 (0)	6 (60.0)	7 (70.0)	8 (80.0)
**Meals (entrée and other items including a beverage)**	18 (4.4)	8 (44.4)	3 (16.7)	0 (0)	16 (89.0)	17 (94.4)
**Exempt items (as listed by CBFAI)**	79 (19.4)	0 (0)	0 (0)	0 (0)	0 (0)	0 (0)
**Total**	407 (100.0)	93 (22.9)	3 (0.7)	131 (32.2)	60 (14.7)	214 (52.5)

a Milk, eggs, nuts, and nut butters are exempt from saturated fat limits.

b Milk (flavored and unflavored) has an increased limit for sugar of 25.5 g per reference amount customarily consumed (RACC).

c Yogurt has an increased sugar limit of 29 g per RACC.

d 100% fruit and vegetable juices and fresh, whole fruits and vegetables are exempt from all limits.

Of the products on CFBAI’s list, 53% (n = 214) did not meet IWG recommendations for 1 or more nutrients to limit (Figure). Two product categories (cheese and cheese products and mixed dishes) did not have any products listed that met the IWG recommendations. Sugar was the most common IWG-recommended nutrient to limit that was not met among the listed products (n = 131, 32.0%). Nearly all (99%) of the products met the recommendations for *trans* fat. Roughly, 23% and 15% of total products failed to meet the recommendations for saturated fat and sodium, respectively. Conversely, all dairy-based desserts and seeds, nuts, and nut butters and spreads met the IWG recommendations in part because of exemptions from nutrients to limit.

**Figure:**
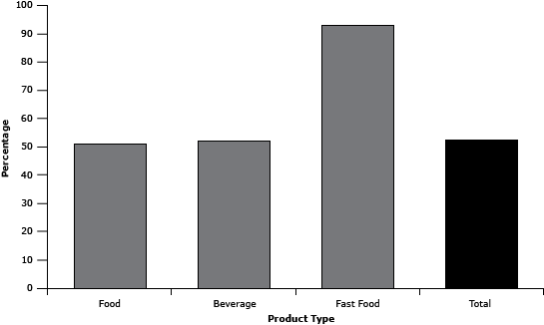
Percentage of food, beverage, fast-food products, and total products on the Children’s Food and Beverage Advertising Initiative’s April 2014 list that exceed the Interagency Working Group’s (IWG’s) recommended nutrients to limit (saturated fat, *trans* fat, sugar, and sodium). **Table:** 

Product	Percentage
Food	51.0
Beverage	52.0
Fast food	93.0
Total	53.0

## Discussion

This is the first study to our knowledge that has evaluated food and beverage products that CFBAI has designated as appropriate for advertisement to children on television since implementation of the new CFBAI uniform nutrition criteria. Our study used dietary recommendations that were drafted jointly by IWG member agencies as directed by Congress in the 2009 Omnibus Appropriation Act (3). These recommendations have not been adopted formally; however, they are the most thorough set of recommendations agreed on by multiple federal agencies regarding food and beverage product advertising on children’s television programs and have been used as an assessment tool in other studies (1,9–11). Both the new CFBAI and IWG recommendations are aligned with the 2010 Dietary Guidelines for Americans and have been developed with input from nutritionists and scientists (3,4); the IWG recommendations are more stringent overall (1). For example, CFBAI sodium restrictions vary from 110 mg or less to 740 mg or less per labeled serving size across product categories, whereas the IWG sodium limits are 210 mg or less and 450 mg or less per labeled serving size for individual items and main dishes or meals, respectively. Additionally, CFBAI offers a range of limits (2 g–25 g of sugar per unit of measure) across product categories. IWG recommendations restrict sugar to 13 g per unit of measure for all food and beverage products.

Our results show that 53% of the CFBAI products approved in April 2014 did not meet IWG recommendations. These findings are consistent with and reveal limited improvement from findings of a study that reported 59% of CFBAI-approved products did not meet similar nutrition guidelines adapted from the National Alliance for Nutrition and Activity’s Model School Nutrition in 2009 (7). We found that of currently CFBAI-approved food products, 23% exceeded the IWG-recommended limit for saturated fat, less than 1% exceeded the limit for *trans* fat, 32% exceeded the limit for sugar, and 15% exceeded the limit for sodium. These findings are similar to those of a study showing that 29%, 0%, 25% and 33% of the 2011 CFBAI-approved products did not meet these same respective IWG recommendations (9).The results reported in this article are conservative, because we addressed only IWG Principle B (nutrients to limit). Other research has found that when IWG Principle A (nutrients to encourage) is applied, the number of products eligible to be advertised on children’s television programs decreases (9,10).

Companies manufacture food and beverage products that meet IWG recommendations; however, these are not the products most heavily marketed to children. Evidence shows that 96% of food and beverage product advertisements (excluding those for restaurants) seen by children on children’s television programs were for products high in nutrients to limit (1). If companies choose to advertise products from the CFBAI’s list of approved food products that meet the IWG recommendations more often than products that do not, children’s exposure to food and beverage advertising could improve substantially. We recommend continued monitoring of child-directed marketing by public health researchers and that the public encourage the food and beverage industry to market their healthiest products to young consumers.

## Appendix

Companies participating in CFBAI as of April 2014 are Burger King Corp, Campbell Soup Company, ConAgra Foods Inc, General Mills Inc, Kellogg Company, Kraft Foods Group Inc, McDonald’s USA, Mondelez Global LLC, PepsiCo Inc, Post Foods, LLC, and Unilever United States. Five participating companies (The Coca-Cola Company, Ferrero USA, Inc, The Hershey Company, Mars, Inc, and Nestle USA) have committed not to engage in child-directed food and beverage product advertising.
